# Unsafe child feces disposal status in Ethiopia: what factors matter? Analysis of pooled data from four demographic and health surveys

**DOI:** 10.1186/s12889-020-08945-6

**Published:** 2020-05-27

**Authors:** Biniyam Sahiledengle

**Affiliations:** School of Health Science, Department of Public Health, Madda Walabu University Goba Referral hospital, Bale-Goba, Ethiopia

**Keywords:** Child feces disposal, Safe management of feces, Stool disposal, Demographic and health survey, Pooled data analysis, Water sanitation and hygiene (WASH), Ethiopia

## Abstract

**Background:**

The only safest way to dispose of a child’s feces is to help the child use a toilet or, for very young children, to put or rinse their feces into a toilet, whereas other methods are considered unsafe. This study aimed to determine the magnitude and factors associated with unsafe child feces disposal in Ethiopia.

**Methods:**

This was a cross-sectional study using pooled data from the four rounds of Demographic and Health Surveys (DHS) conducted in Ethiopia (2000, 2005, 2011, and 2016). Data on child feces disposal practice was collected for all children born during the 5 years preceding survey. Mothers were asked for the youngest child born, “The last time child passed stools, what was done to dispose of the stools?”.Descriptive statistics were computed to illustrate the given data. Multivariable logistic regression was performed to identify factors associated with unsafe child feces disposal.

**Results:**

The pooled dataset contains data for 40,520 children younger than 5 years, male accounts 20,629 (50.9%). Overall, 77.7% (95%CI: 76.3–79.0) of children feces disposed of unsafely. In the multivariable logistic regression model, those mothers whose child was 13–24 months [AOR: 0.68, 95% CI: (0.60–0.78)] and ≥ 25 months [AOR: 0.66, 95% CI: (0.60–0.72)] were lower odds of unsafe child’s feces disposal. Children born into households having two or fewer children were 33% lower [AOR: 0.67, 95% CI: (0.56–0.79)] odds of unsafe child’s feces disposal than their counterparts. The odds of disposing of feces unsafely among households having improved toilet facility was 76% lower [AOR: 0.24, 95% CI: (0.19–0.29)] that of households lacking such facilities. Further, being an urban resident, having improved drinking water facility, a high level of maternal and paternal education, paternal occupational status (work in non-agriculture), and maternal age (25–34 and ≥ 35 years) were factors that associated with lower odds of unsafe child’s feces disposal.

**Conclusions:**

Three in four Ethiopian children feces disposed of unsafely. Household and socio-demographic factors, such as access to improved toilet facility, the child’s age (older age), and both higher maternal and paternal education levels were important factors that significantly associated with lower odds of unsafe child feces disposal.

## Background

Proper disposal of child feces in a toilet or latrine connected to a safe sanitation chain, or helping the child to use a toilet is the only safe method. Otherwise, other disposal methods (feces put/rinsed into a drain or ditch, bush, or thrown into the garbage, buried or left on the ground, and not disposed of) are considered unsafe [1–3].

Unsafe child feces disposal can have a serious impact on children’s health, and there is a growing body of literature demonstrating the link between unsafe child feces disposal and increase risk of diarrheal diseases, environmental enteropathy, and impaired growth [1, 4–13]. Recent studies conducted in Asian and African countries showed that unsafe disposal of children’s feces in the community was strongly associated with increased risk of diarrhea and enteric infections [5–14]. Complementing this finding, a review by Gil et al. found that unsafe disposal of children’s feces associated with a 23% increase in the risk of diarrheal diseases in children [7]. Beyond the immediate impact of diarrheal disease, unsafe disposal of children’s feces can also have long-lasting implications connected with impaired growth [1, 15]; children in households were caregivers reported unsafe feces disposal had significantly greater odds of being wasted [4]. Additionally, evidence from 34 countries found that child feces disposal in an improved toilet was associated with reduced stunting for children under five [15]. A recent study in Bangladesh also reported that young children mouthing soil during play in households with visible feces on their compound had an increased risk of stunting [16].

Despite the evidence to the contrary, the feces of children are less likely to be safely disposed of in a toilet than those of the general population, and poor disposal of children’s feces is drowning the open defecation free (ODF) progress throughout the globe [1–3, 17–21]. According to the United Nations Child Fund (UNICEF) and the World Bank Global Water Practice’s (WSP), over 50% of households with children under age three reported that the feces of their children were unsafely disposed of [3, 22]. Even among households with improved sanitation, unsafe child feces disposal behavior was frequently reported [3, 23, 24]. So far, efforts to combat open defecation (OD) have mainly targeted adults, with only a limited focus on the management of child feces in low-and middle-income countries (LMIC) and how children’s feces are being disposed of, in general, has remained a neglected area of research, policy, and program intervention [2, 3, 6, 15, 25–31]. Still, in many settings, toilets are not designed for or used by children, and young children tend to defecate in environments around the house where susceptible children could be exposed to fecal pathogens [25, 32]. In some cases, parents also discourage children from using a latrine with a squatting slab because they believe that children will dirty the latrine, which possibly endorses open defecation (OD) [13]. Further, there is a widespread wrong perception and cultural beliefs towards child feces management in many communities [2, 33]. It is therefore very important to implement educational interventions to enhance the behavior of the children’s mothers and caregivers towards safe child feces disposal since they are responsible for disposing of their children’s feces and shaping the child’s toilet training. Additionally, exposing mothers to the advantages of potties and educating them on how to potty-train their children as part of sanitation and hygiene programs might lead to improved child feces disposal [15, 28, 30, 33].

At present, irrefutable evidence implied that sanitation for everyone everywhere has been accelerated throughout the globe [34, 35], as part of the overall drive to achieve the Sustainable Development Goal (SDG) and to end OD. Safe disposal of children’s feces is also an essential factor in the achievement of ODF status within communities [1]. In Ethiopia, efforts have been made to create ODF villages through the organized effort of the community by adopting the Community-Led Total Sanitation and Hygiene (CLTSH) approach. For some time now, Ethiopia has recognized unsafe child feces disposal as essential as other sanitation problems so include specific child feces related criteria in ODF verification protocols and national sanitation policies [19, 36, 37]. Despite substantive efforts made at national and regional levels, OD persists in the country and the magnitude of unsafe child feces disposal remains unclear. Therefore, the purpose of this study is to determine the magnitude and factors associated with unsafe child feces disposal in Ethiopia using pooled data from the Ethiopian Demographic and Health Survey (EDHS 2000–2016).

## Methods

### Study design and data source

A was a cross-sectional study. The pooled datasets used in this study were collected from the Ethiopian Demographic and Health Survey (EDHS) conducted in 2000 (EDHS-1), 2005 (EDHS-2), 2011 (EDHS-3), and 2016 (EDHS-4) [38–41]. The EDHS employs a two-stage, stratified, and cluster random sampling technique in order to ensure national representativeness. At the first stage of sampling, enumeration areas (EA) were selected using systematic sampling with probability proportional to size. In the second stage of sampling, a systematic sample of households per EA was selected in all the regions to provide statistically reliable estimates of key demographic and health variables. A representative sample of 11,645 households from 539 clusters (138 in urban areas and 401 in rural areas) in 2000 EDHS [38]; 14,500 households from 540 clusters (145 urban and 395 rural) in 2005 EDHS [39]; 17,817 households from 624 clusters (187 in urban areas and 437 in rural areas) in 2011 EDHS [40], and 16,650 households from 645 clusters (202 in urban areas and 443 in rural areas) in 2016 EDHS [41] were selected for the surveys and the response rates were 99, 98, 94, and 98%, respectively. Details of the survey methodology are described elsewhere [38–41]. The present study included all youngest children under age five living with the mother and mothers were asked about the disposal practice of the last passed feces for the youngest child. All respondents who responded to the outcome variable were included in the analysis for this study.

### Study variables

#### Outcome variable

The outcome variable for this study was “unsafe child feces disposal”. As suggested by the World Health Organization (WHO), unsafe disposal of children’s feces was defined as the disposal of feces in any site other than a latrine. Response categories such as “child used toilet or latrine” and “put/rinsed into toilet or latrine” were combined as “safe disposal” [1].

#### Explanatory variables

The explanatory variables include sex of the child (male, female), age of the child (0–12 months, 13–24 months, ≥ 25 months), maternal age (< 24, 24–34, ≥ 34 years), maternal educational level (no education, primary, secondary, higher), mother’s working status (not working, working), paternal educational level (no education, primary, secondary, higher), partner occupational status (working in agriculture, work in non-agriculture, not working), household size (< 5, ≥ 5), number of children 5 and below (≤ 2, ≥ 3), main floor material (cement, earth), sex of household head (male, female), place of residence (urban, rural), mother’s exposure to media (yes, no), toilet facility (improved, unimproved), sources of drinking water (improve, unimproved) and presence of diarrhea in the last 2 weeks (yes, no) [9, 12, 22, 26, 27].

The variable on media exposure includes exposure to the radio and television. The mothers who were not exposed to radio/television were coded as “no” and those who have frequent exposure were coded as “yes”. Also, the toilet facility and source of drinking water were categorized into ‘improved’ and ‘unimproved’ based on the WHO/ UNICEF JMP for water supply & Sanitation definition [42].

### Operational definitions

**Unsafe child feces disposal**: disposing of child feces in open areas or not disposing of them at all; those left in the open, thrown into the garbage, put/washed/rinsed into open drains, or buried are considered unsafe [1–3].

**Safe child feces disposal**: a child use a toilet or latrine or, for very young children, to put or rinse their feces into a toilet or latrine was regarded as safe [1, 3].

### Statistical analysis

Data from the four waives of EDHS (2000–2016) is used to carry out the analysis. First, data were examined how outcome and explanatory variables were defined in each survey and, if necessary, create new “variables” that are as identical as possible over the survey years. Next, the four datasets were merged into a single data and analyzed using a complex sample analysis, taking into accounts the strata, clusters, and weight variable. A complex sample analysis is a two-step process in SPSS, (1) create a complex sample “*plan file*” after computing a weight variable (*V005*) and (2), run analyses using the plan file through the complex sample package to account for sample design. DHS also strongly recommends that weights be included in any statistical analysis that conducts with DHS data and complex sample command must be considered for analyses of significance testing or a confidence interval (CI) [43]. A detailed explanation of the weighting procedure can be found in the EDHS methodology report [38–41].

Descriptive summaries (weighted frequency and percentage) were used to explain the number of study participants in the analysis. A complex sample binary logistic regression model was employed and presented the crude odds ratio (COR) with 95% CIs to identify the relationship between unsafe child feces disposal and explanatory variables. Those variables with a *p*-value of < 0.25 were then entered into a multivariable logistic regression to control the effect of confounder’s [44]. Finally, significant variables were identified based on the adjusted odds ratio (AOR) with 95% CIs and p-value < 0.05. The multicollinearity effect was assessed with a cut of off point of variation inflation factor (VIF) of greater than ten. To check the correctness of the final formulated model, the Hosmer–Lemeshow test for the overall goodness of fit was used [45]. All statistical analysis was carried out using SPSS version 20.0 (IBM Corp., Armonk, NY, USA).

### Data quality assurance

In all rounds of EDHS, the data collection tools were pretested and data collectors were passes through extensive training. The training consisted of in-class training, biomarker training, and field practice days. Following the field practice, a debriefing session was held with the pretest field staff, and modifications to the questionnaires were made based on lessons drawn from the exercise [38–41]. In this specific paper, I have greatly worked on data quality assurance by cleaning data before performing analysis.

### Ethical consideration

Informed consent was obtained at the beginning of each interview by the EDHS surveyors and data for DHS are publicly available and can be requested from https://dhsprogram.com/data/.

## Results

### Socio-demographic characteristics

Table 1 presents the background characteristics of the children across the entire pooled dataset. In this study, 40,520 children under age five living with the mother were included. Of these, 20,629 (50.9%) of the children were male, a great majority of children (90.0%) were from the rural area, and almost one out of five children had diarrhea in the past 2 weeks before the survey. The mean (standard deviation) age of the child was 28.5 (±17.6) months.

**Table 1:** The characteristics of the respondents in the DHS pooled data 2000–2016, Ethiopia (*n* = 40,520)

Characteristic	Categories	Weighted frequency	Percent
**Child’s characteristics**			
Sex of the child	Male	20,629	50.9
	Female	19,890	49.1
Age of the child	0–12 months	10,040	24.8
	13–24 months	8093	20.0
	≥25 months	22,386	55.2
Diarrhea in the past 2 weeks (*n* = 38,037)	Yes	6616	17.4
	No	31,421	82.6
**Mother’s characteristics**			
Age of mother (in years)	15–24	10,216	25.2
	25–34	20,388	50.3
	> = 35	9916	24.5
Marital status	Married	37,281	92.0
	Divorced/separated	1651	4.1
	Widowed	576	1.4
	Living with a partner	823	2.0
	Single	188	0.5
Mother’s working status (*n* = 40,404)	Not working	21,250	52.6
	Working	19,155	47.4
Mother’s education	No education	30,365	74.9
	Primary	8258	20.4
	Secondary	1527	3.8
	Higher	369	0.9
**Paternal characteristics**			
Partner educational level (*n* = 39,822)	No education	22,254	55.9
	Primary	13,485	33.9
	Secondary	3193	8.0
	Higher	890	2.2
Partner occupational status (*n* = 40,006)	Working in agriculture	32,539	81.3
	Work in non-agriculture	6794	17.0
	Not working	673	1.7
**Household characteristics**			
Household size	Less than 5	9841	24.3
	5 or more	30,679	75.7
Number of children 5 and below	2 or less	33,572	82.9
	3 and above	6948	17.1
Sex of household head	Male	35,364	87.3
	Female	5156	12.7
Place of residence	Urban	4032	10.0
	Rural	36,488	90.0
Main floor material (*n* = 39,595)	Cement	2705	6.8
	Earth	36,890	93.2
**Media exposure**			
Listening radio (*n* = 40,508)	Yes	14,094	34.8
	No	26,414	65.2
Watching TV (*n* = 40,476)	Yes	6369	84.3
	No	34,107	15.7
**Water and sanitation facility**			
Sources of drinking water (*n* = 39,685)	Improved	17,519	44.1
	Unimproved	22,165	55.9
Latrine type (*n* = 39,698)	Improved	4475	11.3
	Unimproved	35,223	88.7
**Survey year**	2000	11,550	28.5
	2005	10,692	26.4
	2011	11,413	28.2
	2016	6864	16.9

### Unsafe child feces disposal

**Fig. 1:**
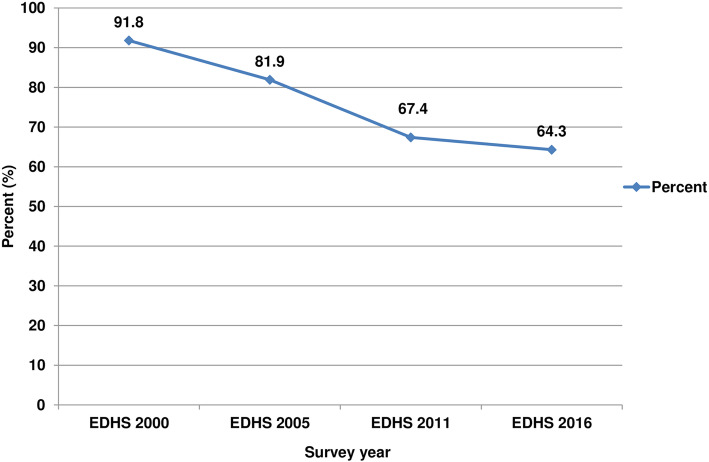
Unsafe child feces disposal in Ethiopia, DHS 2000–2016 (*n* = 40,520)

This study revealed that 77.7% (95%CI: 76.3–79.0) of the children feces in Ethiopia were disposed of unsafely (Table 2). The proportion of unsafe child feces disposal has decreased from 91.8% (95%CI: 90.0–93.3) in the year 2000, to 81.9% (95%CI: 79.4–84.2) in 2005, 67.4% (95%CI: 64.5–70.2) in 2011, and 64.3% (95%CI: 60.4–68.0) in 2016 (Fig. 1). Despite the decline of unsafe child feces disposal over the last 16 years, it is not statistically significant (Additional file 1).

**Table 2:** Weighted child feces disposal practice in Ethiopia, pooled data from DHS 2000–2016 (*n* = 40,520)

Child stool disposal practice	Weighted Frequency	Weighted percent with 95% (CI)
Always use toilet/latrine	831	2.1 (1.8–2.4)
Throw in toilet/latrine	8217	20.3 (19.0–21.6)
Throw outside the dwelling	5158	12.7 (11.6–14.0)
Throw outside the yard	7598	18.8 (17.6–20.0)
Bury in the yard	2088	5.2 (4.5–5.9)
Rinse away	4397	10.9 (10.0–11.8)
Use disposable diapers	402	1.0 (0.8–1.3)
Use washable diapers	1993	4.9 (4.2–5.7)
Not disposed of	7000	17.3 (15.9–18.7)
Other	2837	7.0 (6.3–7.8)
**Overall pooled child stool disposal practice**		
Unsafe ^a^	31,471	77.7 (76.3–79.0)
Safe	9048	22.3 (21.0–23.7)

^a^Unsafe disposal of child feces was defined as disposal of feces in any site other than a sanitary latrine

### Child feces disposal for urban-rural households

**Fig. 2:**
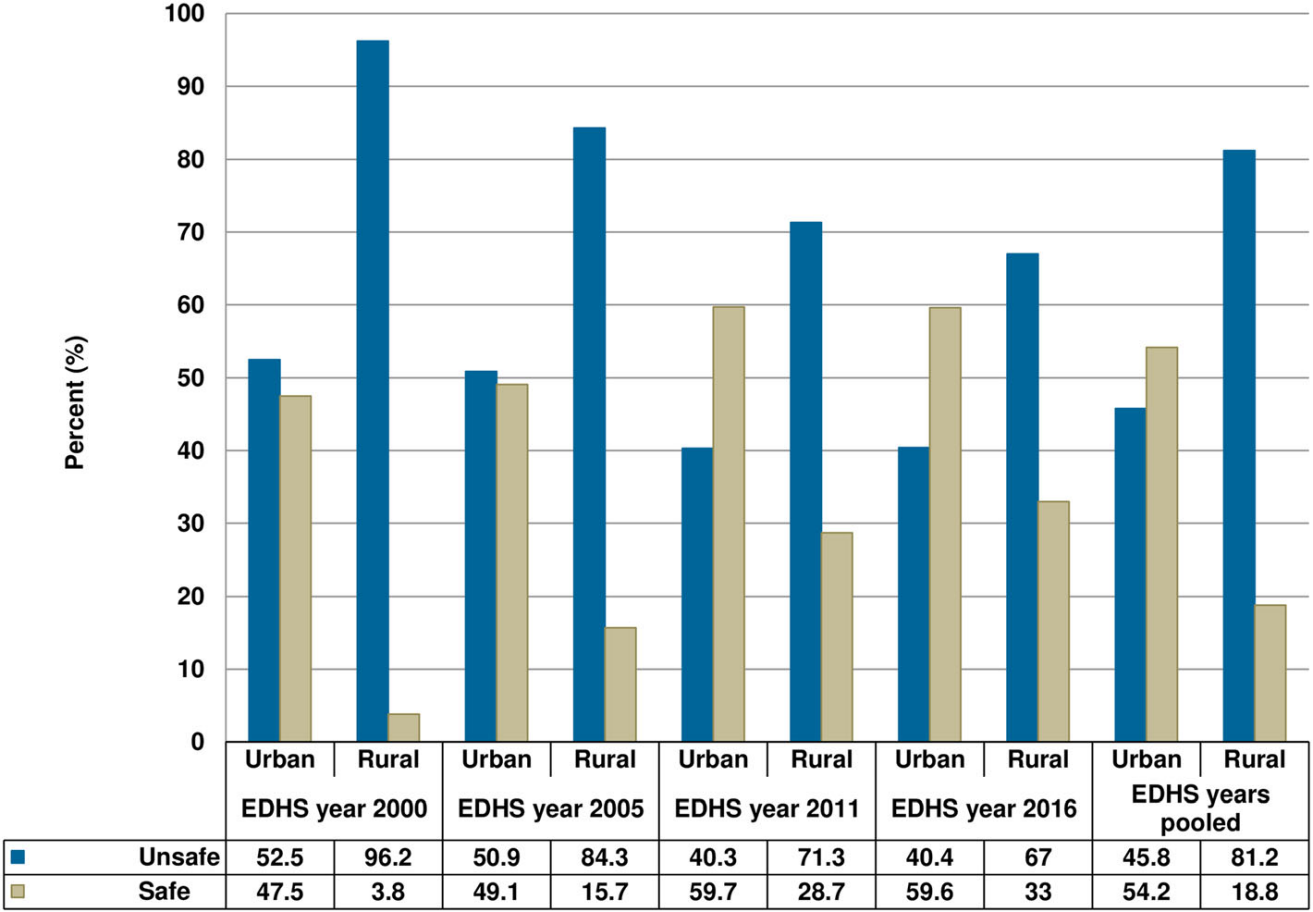
Child feces disposal status among urban and rural households in Ethiopia, DHS 2000–2016 (*n* = 40,520)

**Fig. 3:**
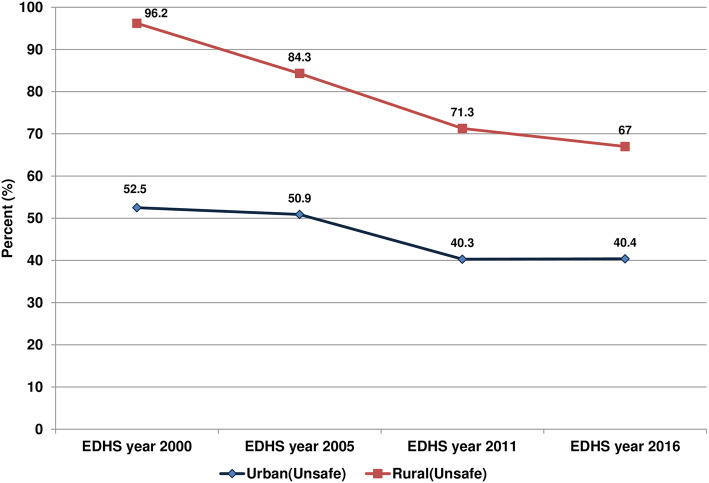
Unsafe feces disposal among urban and rural households in DHS 2000–2016, Ethiopia

Figure 2 and Fig. 3 presents information about child feces disposal in Ethiopia for urban-rural households. Evidence from the pooled data showed, over three fourth of the rural households (81.2%) had unsafe child feces disposal while that is true only for (45.8%) of the urban households. A closer look into the urban-rural households showed that there are wide disparities in unsafe child feces disposal between urban and rural households in all waves of EDHS. The highest level of unsafe child feces disposal was reported among those children from a rural area in the year 2000 (96.2%) and the lowest level was recorded in the year 2011 among urban dwellers (40.3%). The surveys have shown that a slow decrement in unsafe feces disposal in urban-rural households in the past 16 years; from 96.2 to 67% in rural households and from 52.5 to 40.4% in urban households between the year 2000 and 2016.

### Result of bivariate and multivariable analysis

The results of bivariate logistic regression analysis are presented in Table 3. The results indicate that child’s characteristics (age and sex of the child), mother’s characteristics (age and maternal education), paternal characteristics (educational status and occupational status), household characteristics (number of children 5 and below, sex of household head, residence place, and main floor material), media exposure (listening to the radio and watching TV), and WASH-related variables (latrine type and sources of drinking water) were associated with unsafe child feces disposal.

**Table 3:** Bivariate logistic regression result on factors associated with unsafe child feces disposal in Ethiopia DHS 2000–2016, (*n* = 40,520)

Characteristic	Categories	Child stool disposal		COR(95%CI)	***P***-value
		Unsafe (***n*** = 31,471)	Safe (***n*** = 9048)		
**Child’s characteristics**					
Sex of the child	Male	16,202	4427	1.12(1.04–1.18)*	0.00
	Female	15,269	4621	1	
Age of the child	0–12 months	8035	2005	1	
	13–24 months	6064	2029	0.75(0.67–0.83)*	0.00
	≥25 months	17,372	5014	0.86(0.80–0.93)*	
Diarrhea in the past 2 weeks (*n* = 38,038)	Yes	5220	1396	1	
	No	24,194	7228	0.89(0.79–1.00)	0.06
**Mother’s characteristics**					
Age of mother (in years)	15–24	8197	2019	1	
	25–34	15,509	4879	0.78(0.69–0.88)*	0.00
	≥35	7765	2151	0.89(0.78–1.02)	
Mother’s working status (*n* = 40,403)	Not working	16,371	4878	1	
	Working	15,018	4136	1.08(0.96–1.21)	0.18
Mother’s education	No education	25,181	5184	1	
	Primary	5403	2855	0.39(0.34–0.44)*	0.00
	Secondary	779	748	0.21(0.17–0.26)*	
	Higher	108	261	0.09(0.06–0.12)*	
**Paternal characteristics**					
Partner educational level (*n* = 39,825)	No education	18,834	3421	1	
	Primary	9828	3657	0.49(0.43–0.55)*	0.00
	Secondary	1923	1271	0.28(0.23–0.33)*	
	Higher	347	544	0.12(0.09–0.15)*	
Partner occupational status (*n* = 40,006)	Working in agriculture	26,817	5721	1	
	Work in non-agriculture	3839	2955	0.28(0.24–0.32)*	0.00
	Not working	444	229	0.41(0.29–0.59)*	
**Household characteristics**					
Household size	Less than 5	7552	2289	0.93(0.85–1.03)	0.15
	5 or more	23,919	6759	1	
Number of children 5 and below	2 or fewer	25,857	7715	0.79(0.69–0.92)*	0.00
	3 and above	5614	1333	1	
Sex of household head	Male	27,636	7728	1	
	Female	3835	1321	0.81(0.71–0.92)*	0.00
Place of residence	Urban	1847	2185	0.19(0.16–0.24)*	0.00
	Rural	29,625	6863	1	
Main floor material (*n* = 39,595)	Cement	1117	1588	0.17(0.14–0.21)*	0.00
	Earthen floors	29,615	7275	1	
**Media exposure**					
Listening radio (*n* = 40,508)	Yes	9798	4296	0.50(0.45–0.56)*	0.00
	No	21,663	4751	1	
Watching TV (*n* = 40,476)	Yes	3595	2774	0.29(0.25–0.34)*	0.00
	No	27,834	6274	1	
**Water and sanitation facility**					
Sources of drinking water (*n* = 39,685)	Improved	12,582	4937	0.56(0.48–0.64)*	0.00
	Unimproved	18,195	3971	1	
Latrine type (*n* = 39,698)	Improved	2093	2382	0.20(0.17–0.23)*	0.00
	Unimproved	28,696	6527	1	
**Survey year**	2000	10,602	948	1	
	2005	8762	1931	0.41(0.31–0.53)*	0.00
	2011	7697	3716	0.19(0.14–0.24)*	
	2016	4411	2453	0.16(0.12–0.21)*	

Crude odds ratio (COR); *significantly associated *p*-value < 0.05(crude)

In the multivariable logistic regression model, the odds of unsafe child feces disposal were higher [AOR: 1.11, 95%CI: (1.03–1.21)] among households having male children than households having female children. Children aged 13–24 months [AOR: 0.68, 95% CI: (0.60–0.78)] and ≥ 25 months [AOR: 0.66, 95% CI: (0.60–0.72)] were less likely to have their feces disposed unsafely than children age between 0 and 12 months. Lower odds of unsafe child feces disposal was observed among children born to mothers aged 25–34 years [AOR: 0.74, 95%CI: (0.63–0.87)] and ≥ 35 years [AOR: 0.69, 95%CI: (0.57–0.82)] compared to those children born to mothers aged 15–24 years. The odds of unsafe child feces disposal were 35% [AOR: 0.65, 95%CI: (0.55–0.76)] and 27% lower [AOR: 0.73, 95%CI: (0.55–0.96)] in mothers who had primary and secondary education than mothers who had no education, respectively. Likewise, higher paternal educational level and working in non-agriculture were associated with lower odds of unsafe feces disposal (Table 4).

**Table 4:** Multivariable logistic regression result on factors associated with unsafe child feces disposal in Ethiopia DHS, 2000–2016 (*n* = 40,520)

Characteristic	Categories	Child stool disposal		AOR(95%CI)
		Unsafe (***n*** = 31,471)	Safe (***n*** = 9048)	
**Child’s characteristics**				
Sex of the child	Male	16,202	4427	1.11(1.03–1.21)**
	Female	15,269	4621	1
Age of the child	0–12 months	8035	2005	1
	13–24 months	6064	2029	0.68(0.60–0.78)**
	≥25 months	17,372	5014	0.66(0.60–0.72)**
Diarrhea in past 2 weeks (*n* = 38,038)	Yes	5220	1396	1
	No	24,194	7228	1.25(1.11–1.42)**
**Mother’s characteristics**				
Age of mother (in years)	15–24	8197	2019	1
	25–34	15,509	4879	0.74(0.63–0.87)**
	> = 35	7765	2151	0.69(0.57–0.82)**
Mother’s working status (*n* = 40,403)	Not working	16,371	4878	1
	Working	15,018	4136	1.04(0.92–1.17)
Mother’s education	No education	25,181	5184	1
	Primary	5403	2855	0.65(0.55–0.76)**
	Secondary	779	748	0.73(0.55–0.96)**
	Higher	108	261	0.87(0.50–1.53)
**Paternal characteristics**				
Partner educational level (*n* = 39,825)	No education	18,834	3421	1
	Primary	9828	3657	0.74(0.64–0.85)**
	Secondary	1923	1271	0.56(0.46–0.69)**
	Higher	347	544	0.72(0.50–1.04)
Partner occupational status (*n* = 40,006)	Working in agriculture	26,817	5721	1
	Work in non-agriculture	3839	2955	0.74(0.62–0.90)**
	Not working	444	229	0.94(0.61–1.42)
**Household characteristics**				
Household size	Less than 5	7552	2289	1.09(0.96–1.25)
	5 or more	23,919	6759	1
Number of children 5 and below	2 or fewer	25,857	7715	0.67(0.56–0.79)**
	3 and above	5614	1333	1
Sex of household head	Male	27,636	7728	1
	Female	3835	1321	1.00(0.86–1.17)
Place of residence	Urban	1847	2185	0.64(0.49–0.82)**
	Rural	29,625	6863	1
Main floor material (*n* = 39,595)	Cement	1117	1588	0.71(0.54–0.92)**
	Earthen floors	29,615	7275	1
**Media exposure**				
Listening radio (*n* = 40,508)	Yes	9798	4296	0.88(0.76–1.01)
	No	21,663	4751	1
Watching TV (*n* = 40,476)	Yes	3595	2774	0.96(0.72–1.03)
	No	27,834	6274	1
**Water and sanitation facility**				
Sources of drinking water (*n* = 39,685)	Improved	12,582	4937	0.82(0.70–0.94)**
	Unimproved	18,195	3971	1
Latrine type (*n* = 39,698)	Improved	2093	2382	0.24(0.19–0.29)**
	Unimproved	28,696	6527	1
**Survey year**	2000	10,602	948	1
	2005	8762	1931	0.34(0.27–0.43)**
	2011	7697	3716	0.15(0.12–0.19)**
	2016	4411	2453	0.11(0.09–0.14)**

*AOR* adjusted odds ratio; ** significantly associated *p*-value < 0.05(Adjusted)

In this study, the odds of unsafe feces disposal was 36% lower [AOR: 0.64, 95%CI (0.49–0.82)] in households residing in urban areas than households residing in rural areas. The odds of unsafe feces disposal were 33% lower [AOR: 0.67, 95% CI: (0.56–0.79)] among households having two or fewer under 5 children than their counterparts. The odds of disposing of feces unsafely among households having improved drinking water and improved toilet facility were 18% [AOR: 0.82, 95% CI: (0.70–0.94)] and 76% lower [AOR: 0.24, 95% CI: (0.19–0.29)] than that of households lacking such facilities, respectively (Table 4).

The odds of unsafe child feces disposal was 25% higher [AOR: 1.25, 95% CI: (1.11–1.42)] in children without diarrhea compared to children who suffer from diarrhea. From the pooled data, the odds of unsafe feces disposal were 66% [AOR: 0.34, 95% CI:((0.27–0.43)], 85% [AOR: 0.15, 95%CI (0.12–0.19)] and 89% lower [AOR: 0.11, 95%CI: (0.09–0.14)] in EDHS 2005, 2011 and 2016, respectively compared to EDHS conducted in 2000 (Table 4).

## Discussion

The current study aimed to assess the magnitude and factors associated with unsafe child feces disposal in Ethiopia using pooled data from the 2000–16 Ethiopia DHS. The pooled data contained 40,520 children under age five, which were included in the study. Of these, 77.7% of them had an unsafe child’s feces disposal. The study revealed that unsafe child’s feces disposal was less prevalent in households with improved water and toilet facility, those in urban residents, those with older children, those with a high level of maternal and paternal education, and those with lower numbers of under-five children.

The high proportion of unsafe child’s feces disposal found in this study was in line with studies conducted in India (79.0%) [9], 81.4% in Orissa (India) [46], Bangladesh (84%) [4], Malawi (79%) [47], and in Uganda (75%) [48]. The Multiple Indicator Cluster Survey (MICS) reports also showed that more than 50% of households with children under age three in 15 of the 26 locations, particular in Africa, South Asia, and Southeast Asia reported that the feces of their youngest child under age three were not deposited into any kind of improved or unimproved toilet or latrine i.e., they were unsafely disposed of [3]. In this study, a considerable number of children feces disposed of in the open field, which may put children at risk of fecal exposure and diarrheal illness. In support of this, a study in Bangladesh explores the link between unsafe feces disposal in the residential compound and increase the risk of fecal exposure [49]. Bawankule et al. (2017) also found that unsafe disposal of children’s feces even in the neighborhood was associated with a higher risk of diarrhea in children. A review showed that diarrheal diseases were prevalent in areas where poor hygiene and sanitation is widespread [7].

Although the decline of unsafe child feces disposal over the last 16 years is not statistically significant, there was a modest drop of unsafe feces disposal in Ethiopia from 91.8% in the year 2000 to 64.3% in 2016. This is less than 30% in 16 years or about 2% per year, which is very low and it can that signify the problem of unsafe child feces disposal remain in the country. This finding, therefore, embodies an important message for the ongoing WASH, CLTS, and other sanitation-related projects in the country. First, interventions that encourage children to use the latrine directly may be potentially beneficial to improve the current practice. Second, enhancing the behavior of the children’s mothers/caregivers is essential, since in many cases they are responsible for disposing of their children’s feces and shaping the child’s toilet training. Third, access to a latrine is a necessary condition to have a positive effect on the reduction of unsafe feces disposal [50, 51].

In the multivariable logistic regression analysis, the odds of unsafe child feces disposal were lower in mothers who had primary and secondary education than mothers who had no formal education. These observations are quite as expected because less-educated parents are more likely to be unaware of the health risks associated with unsafe excreta disposal and therefore practice unsafe disposal [52]. This finding is in accordance with other studies done in Kenya [53], and India [54].

Consistent with studies in Bangladesh [55, 56], Malawi [57], and Cambodia [27], women with younger children were more likely to report unsafely dispose of their children’s feces compared with those with older children. This association can be satisfactorily explained by the fact that a shift in safe disposal is usually seen as they get older [19]. To overcome unsafe feces disposal among young children, Hussain et al. suggested four behaviors that should be promoted in a child potty behavior: 1) acquisition of a potty, 2) potty training, 3) regular emptying of the potty into a latrine, and 4) cleaning and maintenance for continued use [51]. In support of this suggestion, studies from Nigeria [28] and Bangladesh [51] showed that child defecation in potties was strongly associated with safe feces disposal. This study further revealed that child feces disposal was associated with maternal age, media exposure, and toilet/latrine access, which is generally consistent with other studies conducted elsewhere [9, 27, 51, 54, 58]. Again, the place of residence was another factor associated with unsafe child feces disposal. The odds of practicing unsafe child feces disposal were significantly lower among urban residents. This coincides with other similar reports [3, 53]. Somewhat surprisingly, the association between unsafe child feces disposal and decreased odds of diarrhea diseases was not detected in the present study. However, several studies done in low-income settings, such as Nepal [5], Indonesia [6], Thailand [8], India [9], Burkina Faso [13], and Nigeria [59] reported the association between unsafe feces disposal and increased odds of childhood diarrhea.

### Limitations of the study

This study has several limitations. First, the study suffers from the disadvantages of a cross-sectional study; the temporal relationship between the outcome and explanatory variables could not be established. Second, the study did not record how feces were transported for disposal in study households. This would have added an understanding of the relationship between unsafe child feces disposal and transportation mechanisms. Third, reporting bias is likely to over-report child feces disposal behavior. Fourth, the study may be susceptible to recall bias, as the data dealt with reported practices rather than direct observation of the actual practice. Fifth, the measurement of the prevalence of diarrhea in all EDHS is based on a 2 weeks recall period, which may introduce a recall and reporting bias in childhood diarrhea prevalence. Sixth, the study didn’t use multilevel analysis which is the ideal alternative to address nested data. Therefore, the associations that were found in the multivariable analysis should thus be interpreted cautiously. Finally, despite there were similar trends for many of the countries in the practice of child feces disposal, I would suggest caution against applying the results to countries located in other regions of the world, as cultural differences may affect child stool disposal practices.

## Conclusion

Three in four Ethiopian children feces disposed of unsafely. Unsafe child’s feces disposal is less prevalent among households that had improved water and toilet facility, those residing in urban areas, those with older children, those with a high level of maternal and paternal education, and those with a lower number of under-five children. The finding highlighted, there is a need for more attention to be paid to curb the significant burden of unsafe child feces disposal in Ethiopia. It is also essential to explore opportunities to integrate child feces management into existing sanitation and hygiene efforts. Moreover, child feces management interventions must consider sanitation coverage as well as behavioral changes, such as efforts to change the behavior of mothers that encourage cleaning children after defecation, potty training at an early age, and using proper methods to transport children feces to a sanitation facility.

## Supplementary information

**Additional file 1.** Unsafe child feces disposal characteristics of the households in DHS 2000, 2005, 2011, and 2016, Ethiopia.

## Data Availability

The dataset was demanded and retrieved from the DHS website https://dhsprogram.com after formal online registration and submission of the project title and detail project description.

## Abbreviations

AOR: Adjusted odds ratio
CI: Confidence interval
CLTS: Community-Led Total Sanitation
COR: Crude odds ratio
DHS: Health and demographic surveys
EDHS: Ethiopian Health and demographic surveys
ODF: Open defecation free
OD: Open defecation
SDGs: Sustainable Development Goals
SPSS: Statistical Package for Social Sciences
VIF: Variance inflation factor
WHO: World Health Organization

## References

[1] World Health Organization (WHO): Guidelines on sanitation and health. 2018, Licence: CC BY-NC-SA 3.0 IGO. Genvea. https://apps.who.int/iris/bitstream/handle/10665/274939/9789241514705-eng.pdf?ua=1. Accessed 13 Apr 2020.

[2] Bain R, Luyendijk R (2015). Are burial or disposal with garbage safe forms of child faeces disposal? An expert consultation. Waterlines..

[3] Rand EC, Loughnan L, Maule L, Reese H (2015). Management of child feces: current disposal practices. Water and Sanitation Program: Research Brief (June).

[4] George CM, Oldja L, Biswas S, Perin J, Sack RB, Ahmed S, Shahnaij M, Haque R, Parvin T, Azmi IJ (2016). Unsafe child feces disposal is associated with environmental enteropathy and impaired growth. J Pediatr.

[5] Lamichhane P, Sharma A, Mahal A (2018). Does safe disposal of child faeces matter? An assessment of access to improved sanitation and child faeces disposal behaviour and diarrhoea in rural Nepal. Int Health.

[6] Cronin A, Sebayang S, Torlesse H, Nandy R (2016). Association of safe disposal of child feces and reported diarrhea in Indonesia: need for stronger focus on a neglected risk. Int J Environ Res Public Health.

[7] Gil A, Lanata C, Kleinau E, Penny M (2004). Children’s feces disposal practices in developing countries and interventions to prevent diarrheal diseases: a literature review. Environmental Health Project.

[8] Wilunda C, Panza A (2009). Factors associated with diarrhea among children less than 5 years old in Thailand: a secondary analysis of Thailand multiple indicator cluster survey 2006. J Health Res.

[9] Bawankule R, Singh A, Kumar K, Pedgaonkar S (2017). Disposal of children’s stools and its association with childhood diarrhea in India. BMC Public Health.

[10] Traore E, Cousens S, Curtis V, Mertens T, Tall F, Traore A, Kanki B, Diallo I, Rochereau A, Chiron J (1994). Child defecation behaviour, stool disposal practices, and childhood diarrhoea in Burkina Faso: results from a case-control study. J Epidemiol Community Health.

[11] Mihrete TS, Alemie GA, Teferra AS (2014). Determinants of childhood diarrhea among underfive children in Benishangul Gumuz regional state, north West Ethiopia. BMC Pediatr.

[12] Baltazar JC, Solon FS (1989). Disposal of faeces of children under two years old and diarrhoea incidence: a case-control study. Int J Epidemiol.

[13] Curtis V, Schmidt W, Luby S, Florez R, Touré O, Biran A (2011). Hygiene: new hopes, new horizons. Lancet Infect Dis.

[14] Roy E, Hasan KZ, Haque R, Haque AF, Siddique A, Sack RB (2011). Patterns and risk factors for helminthiasis in rural children aged under 2 in Bangladesh. South Afr J Child Health.

[15] Bauza V, Guest JS (2017). The effect of young children's faeces disposal practices on child growth: evidence from 34 countries. Tropical Med Int Health.

[16] George CM, Oldja L, Biswas S, Perin J, Lee GO, Kosek M (2015). Geophagy is associated with environmental enteropathy and stunting in children in rural Bangladesh. Am J Trop Med Hyg.

[17] Walker CLF, Perin J, Aryee MJ, Boschi-Pinto C, Black RE (2012). Diarrhea incidence in low-and middle-income countries in 1990 and 2010: a systematic review. BMC Public Health.

[18] Majorin F, Torondel B, Routray P, Rout M, Clasen T (2017). Identifying potential sources of exposure along the child feces management pathway: a cross-sectional study among urban slums in Odisha, India. Am J Trop Med Hygiene..

[19] United Nations Children's Fund: Child feces disposal in Ethiopia. 2014. Available from: https://www.wsp.org/sites/wsp.org/files/publications/WSP-Ethiopia-CFD-Profile.pdf. Accessed 6 Apr 2020.

[20] WHO/UNICEF Joint Water Supply Sanitation Monitoring Programme (2014). Progress on drinking water and sanitation: 2014 Update: World Health Organization.

[21] Pasteur K (2017). Keeping track: CLTS monitoring, certification and verification.

[22] Markovitz AR, Goldstick JE, Levy K, Cevallos W, Mukherjee B, Trostle JA, Eisenberg JN (2012). Where science meets policy: comparing longitudinal and cross-sectional designs to address diarrhoeal disease burden in the developing world. Int J Epidemiol.

[23] Sahiledengle B (2019). Prevalence and associated factors of safe and improved infant and young children stool disposal in Ethiopia: evidence from demographic and health survey. BMC Public Health.

[24] Majorin F, Nagel CL, Torondel B, Routray P, Rout M, Clasen TF (2019). Determinants of disposal of child faeces in latrines in urban slums of Odisha, India: a cross-sectional study. Trans R Soc Trop Med Hyg.

[25] Lanata CF, Huttly SR, Yeager BA (1998). Diarrhea: whose feces matter? Reflections from studies in a Peruvian shanty town. Pediatr Infect Dis J.

[26] Azage M, Haile D (2015). Factors associated with safe child feces disposal practices in Ethiopia: evidence from demographic and health survey. Arch Public Health.

[27] Miller-Petrie MK, Voigt L, McLennan L, Cairncross S, Jenkins MW (2016). Infant and young child feces management and enabling products for their hygienic collection, transport, and disposal in Cambodia. Am J Trop Med Hygiene.

[28] Jinadu M, Adegbenro C, Esmai A, Ojo A, Oyeleye B (2007). Health promotion intervention for hygienic disposal of children's faeces in a rural area of Nigeria. Health Educ J.

[29] Jinadu MK, Esmai OA, Adegbenro CA (2004). Disposal of children's faeces and implications for the control of childhood diarrhoea. J R Soc Promot Heal.

[30] Morita T, Godfrey S, George CM (2016). Systematic review of evidence on the effectiveness of safe child faeces disposal interventions. Tropical Med Int Health.

[31] Mertens T, Jaffar S, Fernando M, Cousens S, Feachem R (1992). Excreta disposal behaviour and latrine ownership in relation to the risk of childhood diarrhoea in Sri Lanka. Int J Epidemiol.

[32] Brown J, Cairncross S, Ensink JH (2013). Water, sanitation, hygiene and enteric infections in children. Arch Dis Child.

[33] Chebet JJ, Kilungo A, Alaofè H, Malebo H, Katani S, Nichter M (2020). Local perceptions, cultural beliefs, practices and changing perspectives of handling infant feces: a case study in a rural Geita District, North-Western Tanzania. Int J Environ Res Public Health.

[34] World Health Organization and UNICEF (2017). Progress on drinking water, sanitation and hygiene: 2017 update and SDG baselines.

[35] UN Vows to Eliminate Open Defecation by 2025. Available from: https://ourworld.unu.edu/en/un-vows-to-eliminate-open-defecation-by-2025. Accessed 4 May 2020.

[36] Federal Democratic Republic of Ethiopia Ministry of Health (2005). National Hygiene and sanitation strategy to enable 100% adoption of improved hygiene and sanitation.

[37] Ministry of Health Ethiopia: Community Led Sanitation and Hygiene (LTSH) verification and certification protocol. Addis Ababa, Ethiopia: Federal Democratic Republic of Ethiopia Ministry of Health.

[38] Central Statistical Authority [Ethiopia] and ORC Macro (2001). Ethiopia Demographic and Health Survey 2000.

[39] Central Statistical Agency [Ethiopia] and ORC Macro. Ethiopia Demographic and Health Survey 2005. Addis Ababa: Central Statistical Agency/ Ethiopia and ORC Macro; 2006.

[40] Central Statistical Agency [Ethiopia] and ICF International (2012). Ethiopia Demographic and Health Survey 2011.

[41] CSA I: Central statistical agency (CSA) [Ethiopia] and ICF. Ethiopia demographic and health survey, Addis Ababa, Ethiopia and Calverton, Maryland, USA 2016.

[42] World Health Organization (WHO) and UNICEF (2006). Core questions on drinking water and sanitation for household surveys. World Health Organization and UNICEF.

[43] IBM: IBM SPSS Complex Samples 22. Armonk, NY 10504–1785. U.S.A. 2013.

[44] Vittinghoff E, Glidden DV, Shiboski SC, McCulloch CE (2012). Logistic regression. InRegression methods in biostatistics.

[45] Hosmer Jr DW. Lemeshow S. Sturdivant RX: Applied logistic regression: John Wiley & Sons. 2013.

[46] Majorin F, Freeman MC, Barnard S, Routray P, Boisson S, Clasen T (2014). Child feces disposal practices in rural Orissa: a cross sectional study. PLoS One.

[47] United Nations Children's Fund: Child feces disposal in Malawi. 2014. Available from: https://www.wsp.org/sites/wsp.org/files/publications/WSP-Malawi-CFD-Profile.pdf. Accessed 10 May 2020.

[48] United Nations Children's Fund: Child feces disposal in Uganda. 2014. Available from: http://www.wsp.org/sites/wsp.org/files/publications/WSP-Uganda-CFD-Profile.pdf. Accessed 10 May 2020.

[49] Kwong LH, Ercumen A, Pickering AJ, Unicomb L, Davis J, Luby SP (2016). Hand-and object- mouthing of rural Bangladeshi children 3–18 months old. Int J Environ Res Public Health.

[50] Phaswana-Mafuya N, Shukla N (2005). Factors that could motivate people to adopt safe hygienic practices in the eastern Cape Province, South Africa. Afr Health Sci.

[51] Hussain F, Luby SP, Unicomb L, Leontsini E, Naushin T, Buckland AJ, Winch PJ (2017). Assessment of the acceptability and feasibility of child potties for safe child feces disposal in rural Bangladesh. Am J Trop Med Hygiene.

[52] Dreibelbis R, Winch PJ, Leontsini E, Hulland KR, Ram PK, Unicomb L (2013). The integrated behavioural model for water, sanitation, and hygiene: a systematic review of behavioural models and a framework for designing and evaluating behaviour change interventions in infrastructure-restricted settings. BMC Public Health.

[53] United Nations Children's Fund: Child feces disposal in Kenya. 2014. Available from: https://www.wsp.org/sites/wsp/files/publications/WSP-Kenya-CFD-Profile.pdf. Accessed 10 May 2020.

[54] Preeti P, Sahoo SK, Biswas D, Dasgupta A (2016). Unsafe disposal of child faeces: a community-based study in a rural block in West Bengal, India. J Prev Med Public Health.

[55] Islam M, Ercumen A, Ashraf S, Rahman M, Shoab AK, Luby SP, Unicomb L (2018). Unsafe disposal of feces of children< 3 years among households with latrine access in rural Bangladesh: association with household characteristics, fly presence and child diarrhea. PLoS One.

[56] Sultana R, Mondal UK, Rimi NA, Unicomb L, Winch PJ, Nahar N (2013). al. E: an improved tool for household faeces management in rural Bangladeshi communities. Tropical Med Int Health.

[57] Nkoka O (2020). Correlates of appropriate disposal of children’s stools in Malawi: a multilevel analysis. BMC Public Health.

[58] Freeman MC, Majorin F, Boisson S, Routray P, Torondel B, Clasen T (2016). The impact of a rural sanitation programme on safe disposal of child faeces: a cluster randomised trial in Odisha, India. Trans R Soc Trop Med Hyg.

[59] Aluko O, Afolabi O, Olaoye E, Adebayo A, Oyetola S, Abegunde O (2017). The management of the faeces passed by under five children: an exploratory, crosssectional research in an urban community in Southwest Nigeria. BMC Public Health.

