# Supplementary material for: Unsafe child feces disposal status in Ethiopia: what factors matter? Analysis of pooled data from four demographic and health surveys
Source: BMC Public Health. 2020 May 27;20:800. doi: 10.1186/s12889-020-08945-6 (PMC7254708; doi:10.1186/s12889-020-08945-6)
Supplement: Supplementary file 1 — Additional file 1. Unsafe child feces disposal characteristics of the households in DHS 2000, 2005, 2011, and 2016, Ethiopia. [file 12889_2020_8945_MOESM1_ESM.docx]

**Table 1: Unsafe child feces disposal characteristics of the households in DHS 2000, 2005, 2011, and 2016, Ethiopia**

| **Survey year** | **Unsafe child feces disposal** | | |
| --- | --- | --- | --- |
|  | **Frequency*** | **Percent** | **95%CI** |
| EDHS 2000, n= 11,550 | 10,602 | 91.8 | 90.0-93.3 |
| EDHS 2005, n= 10,693 | 8,762 | 81.9 | 79.4-84.2 |
| EDHS 2011, n= 11,413 | 7,697 | 67.4 | 64.5-70.2 |
| EDHS 2016, n= 6,864 | 4,411 | 64.3 | 60.4-68.0 |
| **EDHS (2000-2016),**  n=40,520 | 31,452 | 77.7 | 76.3-79.0 |

***** weighted frequency
